# Orphan nuclear receptors as regulators of intratumoral androgen biosynthesis in castration-resistant prostate cancer

**DOI:** 10.1038/s41388-021-01737-1

**Published:** 2021-03-09

**Authors:** Jianfu Zhou, Yuliang Wang, Dinglan Wu, Shusheng Wang, Zhiqiang Chen, Songtao Xiang, Franky Leung Chan

**Affiliations:** 1 Department of Urology, The Second Affiliated Hospital of Guangzhou University of Chinese Medicine, Guangzhou, China; 2 The Second Clinical College, Guangzhou University of Chinese Medicine, Guangzhou, China; 3 School of Biomedical Sciences, Faculty of Medicine, The Chinese University of Hong Kong, Hong Kong, China; 4 Shenzhen Key Laboratory of Viral Oncology, The Clinical Innovation & Research Center, Shenzhen Hospital, Southern Medical University, Shenzhen, China

**Keywords:** Tumour biomarkers, Prostate cancer

## Abstract

Castration-resistant prostate cancer (CRPC) almost invariably occurs after androgen-deprivation therapy (ADT) for the advanced metastatic disease. It is generally believed that among multiple mechanisms and signaling pathways, CRPC is significantly driven by the reactivation of androgen receptor (AR) signaling in ADT-treated patients with castrate levels of androgen, partially at least mediated by the androgen biosynthesis within the tumor, also known as intratumoral or intraprostatic androgen biosynthesis. Steroidogenic enzymes, such as CYP11A1, CYP17A1, HSD3B1, AKR1C3 and SRD5A, are essential to catalyze the conversion of the initial substrate cholesterol into potent androgens that confers the CRPC progression. Accumulating evidences indicate that many steroidogenic enzymes are upregulated in the progression setting; however, little is known about the dysregulation of these enzymes in CRPC. Orphan nuclear receptors (ONRs) are members of the nuclear receptor superfamily, of which endogenous physiological ligands are unknown and which are constitutively active independent of any physiological ligands. Studies have validated that besides AR, ONRs could be the potential therapeutic targets for prostate cancer, particularly the lethal CRPC progression. Early studies reveal that ONRs play crucial roles in the transcriptional regulation of steroidogenic enzyme genes. Notably, we and others show that three distinct ONRs, including liver receptor homolog-1 (LRH-1, *NR5A2*), steroidogenic factor 1 (SF-1, AD4BP, *NR5A1*) and estrogen-related receptor α (ERRα, *NR3B1*), can contribute to the CRPC progression by promotion of the intratumoral androgen synthesis via their direct transcriptional regulation on multiple steroidogenic enzymes. This review presents an overview of the current understanding on the intratumoral androgen biosynthesis in CRPC, with a special focus on the emerging roles of ONRs in this process.

## Introduction

Prostate cancer is the most frequently diagnosed malignancy among males in majority of economically developed countries, and is the second most common cancer in men worldwide [1, 2]. The growth and survival of prostate cancer cells are sustained by androgens through the activation of androgen receptor (AR) and its mediated signalings. Thus, androgens, particularly testosterone (T) and dihydrotestosterone (DHT), which serve as the major endogenous ligands of AR, are the key drivers for both the initiation and progression of prostate cancer. Androgen-deprivation therapy (ADT) or hormone therapy, with a primary aim of depletion of gonadal T and achieved by either medical or surgical castration with or without combination of antiandrogen, has been conventionally used as the standard upfront treatment for locally advanced and metastatic prostate cancer. Unfortunately, the response is usually transient and almost all patients inevitably relapse with progression to the aggressive and fatal castration-resistant prostate cancer (CRPC) [3]. Although not fully understood, multiple and interconnected mechanisms may be involved in castration resistance [4], including intratumoral androgen biosynthesis, AR pathway hypersensitivity due to AR gene amplification, AR activation (often mediated by AR mutations) by noncognate ligands such as corticosteroids or even antiandrogens, increased AR transcription activity mediated by oncogenic growth factor-activated signal pathways, expression of variant AR isoforms (e.g., AR-V7) that are ligand-independent, activation of alternative AR-independent or AR-bypass pathways, and selection of pre-existing prostate cancer stem cells [5, 6]. Among these, the intratumoral or intraprostatic androgen biosynthesis and persistent AR signaling are regarded as the key factors responsible for the progression of CRPC [7]. In this review, we update the current understanding of intratumoral androgen biosynthesis in CRPC, with a particular focus on the emerging roles of ligand-independent orphan nuclear receptors (ONRs) involved in this process.

## Evidences of intratumoral androgen biosynthesis as a key driver in CRPC

It has been well-characterized that DHT is much more potent than T to activate AR, and is the main androgen bound to AR in the nuclei of prostatic cells. Although the presence of intratumoral DHT was first noted over 30 years ago in patients relapsed from orchiectomy or estrogen therapy [8], the most supportive evidence that intratumoral androgen biosynthesis acting as a key driving force in CRPC progression is the survival benefit conferred by the recent clinical use of the key steroidogenic enzyme CYP17A1 inhibitor abiraterone acetate as well as the potent AR antagonist enzalutamide [9–12]. Early study in men with CRPC and intact prostates reported that intraprostatic DHT levels in a small subset of patients were increased relative to those men immediately after castration, although these findings were not interpreted as a supportive evidence for the increased androgen biosynthesis within tumors [8]. By radioimmunoassay or more sensitive mass spectrometry methods, both T and DHT are detected in recurrent prostate cancer tissues [13, 14]. Further analysis reveals that higher levels of T and DHT are detected in primary prostate cancers as compared with paired benign prostate tissues; and levels of T and DHT as measured in the castration-resistant metastases are much higher than those in the non-prostatic control tissues [15]. These results also indicate that residual T levels of 0.2–2.94 ng/g and DHT levels of 0.36–2.19 ng/g, as measured in clinical tissues from CRPC patients, are sufficient to activate AR, stimulate AR-regulated genes and enable tumor cell growth and survival [13–15]. Therefore, understanding the source and regulation of androgen biosynthesis is critical to the development of novel effective therapies for better management of CRPC.

## Pathways and steroidogenic enzymes mediating androgen biosynthesis in CRPC

**Fig. 1:**
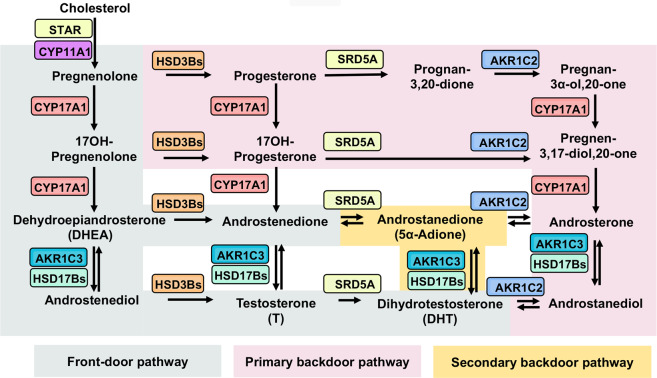
Recognized pathways of androgen biosynthesis in prostate cancer. Three potential pathways currently exist and function in CRPC that may confer increased levels of androgen biosynthesis within the tumor through the sequential actions of steroidogenic enzymes that are normally active in the testes and adrenal glands. Cholesterol is converted to pregnenolone by the action of STAR and CYP11A1. In the front-door (canonical or classical) pathway (greyish green), characterized by the necessity of testosterone (T) as an essential precursor that generate DHT, pregnenolone is converted to dehydroepiandrosterone (DHEA) by the sequential hydroxylase and lyase activity of CYP17A1. DHEA (from intrinsic or adrenal) is then acted on by HSD3B to yield androstenedione or by HSD17B3 (or AKR1C3) to yield androstenediol, which are subsequently converted to T, followed by its 5α-reduction to dihydrotestosterone (DHT) by 5α-reductases (SRD5As). On the other hand, the backdoor pathways refer to use of distinct substrates and enzymatic reactions to synthesize DHT bypassing T as intermediate. In the primary backdoor pathway (pink), the progesterone intermediates are 5α- and 3α-reduced by SRD5As and AKR1C2 before the lyase activity of CYP17A1, forming the androsterone and then to androstanediol by HSD17Bs (or AKR1C3) to generate DHT. In the secondary backdoor (5α-Adione) pathway (yellow), androstenedione as produced in the classical pathway is converted to 5α-androstenedione (5α-Adione) by SRD5As instead of conversion to T, and then to DHT by HSD17Bs (or AKR1C3). The necessary steroidogenic enzymes (gene names) catalyzing different steps of androgen biosynthesis are colour-coded across the three pathways. (STAR = steroidogenic acute regulatory protein; CYP11A1 = cholesterol side-chain cleavage enzyme; CYP17A1 = steroid 17α-monooxygenase; AKR1C3 = aldo-keto reductase 1C3; HSD17Bs = 17B-hydroxysteroid dehydrogenases; HSD3Bs = 3β-hydroxysteroid dehydrogenases; SRD5As = steroid 5α-reductase; AKR1C2 = aldo-keto reductase 1C2.).

Evidences from the past decade indicate that prostate cancer tissues, especially from CRPC patients, express a spectrum of steroidogenic enzymes responsible for additional catalyzing steps for androgen biosynthesis beyond the canonical physiological conversion of T to DHT [16]. It is currently recognized that up to three potential synthetic pathways exist that might lead to the increased levels of androgen biosynthesis within the tumor in CRPC [17–19]. The front-door or classical pathway, which involves the canonical physiological production of T within the testis de novo from cholesterol or converted from circulating adrenal androgen precursors, is characterized by the necessity of T as an essential precursor that generates DHT. On the other hand, the primary and secondary backdoor pathways utilize distinct intermediate substrates (progesterone → → androstanediol → DHT or DHEA → → 5α-Adione → DHT) and steroidogenic enzymes to synthesize DHT bypassing T as the intermediate. A general outline of the front-door and backdoor steroidogenic pathways is illustrated in Fig. 1.

Accumulating evidences show that CRPC tissues as well as xenograft models of CRPC exhibit upregulated expressions of multiple key steroidogenic enzymes responsible for the androgen biosynthesis. In patients with CRPC, dehydroepiandrosterone (DHEA) of adrenal origin and its sulfated derivative (DHEA-S) are implicated as the predominant precursors of T [20]. Compared with primary prostate tumors, castration-resistant metastases displayed alterations in steroidogenic enzyme genes, including up-regulated expression of *CYP17A1, HSD3B1*, and *HSD17B3* and down-regulated expression of *SRD5A2* [15]. Of note, the reduced expression of *SRD5A2* in CRPC is consistent with previous observation that a marked reversal in the ratio of T to DHT exists in the CRPC tissue as compared to the primary prostate tissues [13, 15]. Indeed, although DHT is more potent than T in activating AR, previous kinetic experiments have revealed that T at high concentrations interacts with AR similarly to DHT [21], and 1.6-fold to 1.9-fold increases in T as compared with DHT are sufficient to achieve comparable prostate regrowth [22]. One study conducted primarily in LNCaP-CRPC xenograft model indicates that expressions of the enzymes required for de novo androgen biosynthesis, including CYP11A1, CYP17A1 and AKR1C3, are increased in castration-resistant sublines. Moreover, we and others show that several major steroidogenic enzyme genes involved in androgen biosynthesis (such as steroidogenic acute regulatory gene *STAR, CYP11A1, HSD3B2, CYP17A1* and *AKR1C3*) exhibits upregulated expressions in castration-relapse VCaP xenograft model (VCaP-CRPC), and the expressions of *CYP17A1* and *AKR1C3* are further increased upon treatment with CYP17A1 inhibitor abiraterone [16, 23, 24]. The use of ex vivo radiotracing assays coupled to HPLC/MS detection demonstrates that CRPC tumors are capable of de novo conversion of [^14^C]-acetic acid to DHT and uptake of [^3^H]-progesterone to steroid precursors of DHT, suggesting that de novo androgen biosynthesis may be a driving force leading to CRPC progression following castration [25]. Another study shows that *CYP17A1* and *HSD3B1* mRNA levels are extremely low in locally recurrent CRPC, whereas enzymes that convert androstenedione to T (AKR1C3) and T to DHT (SRD5A1) are abundantly expressed. These results implicate that the enhanced production of adrenal androgens and intratumoral de novo androgen biosynthesis in a subset of CRPC tumors may require additional suppression of intratumoral AKR1C3 or SRD5A1 activity in order to reduce the conversion of adrenal steroid precursors to the active T and/or DHT and consequently AR pathway activation [26]. Furthermore, a gain-of-function mutation of *HSD3B1* [3βHSD1(367T)], a key enzyme regulating the conversion of DHEA via the 5α-androstanedione (5α-dione) pathway to DHT, is detected in CRPC, and the mutant does not affect the catalytic activity but renders the enzyme resistant to ubiquitination and degradation, hence leading to increased DHT level and resulting in AR reactivation [27]. Together, CRPC tumors are characterized by a number of alterations in steroidogenic enzyme gene expression that are consistent with either mediating conversion of adrenal androgen precursors to DHT, or promoting de novo biosynthesis of androgens from cholesterol precursors. Current treatments for CRPC with specific steroidogenic enzyme inhibitors, such as the CYP17A1 inhibitor abiraterone acetate, are insufficient to stop the progression to the lethal form of the metastatic disease [16]. Thus, targeting the upstream factors involved in the regulation of expression of steroidogenic enzymes and exploration of the mechanisms via which intratumoral androgen biosynthesis is initiated and maintained represent an attractive and potential novel strategy for the management of CRPC. Indeed, many early studies have validated that transcriptional regulation of human steroidogenic enzyme genes is responsible for the control of steroid hormone biosynthesis in maintaining various physiological processes [28, 29].

## Involvement and pathogenesis of orphan nuclear receptors in CRPC

Orphan nuclear receptors (ONRs) are members of the nuclear receptor (NR) superfamily, and are so named either because their endogenous physiological ligands are unknown or they are constitutively active independent of any physiological ligands [30]. The common structure of ONRs contains four typical functional domains: (1) N-terminal activation domain [or transcriptional activation function-1 site (AF-1)], (2) DNA-binding domain (DBD), (3) hinge region, (4) ligand-binding domain (LBD) and C-terminal activation domain (AF-2) that interacts with co-regulators. ONRs share two highly conserved domain structures, DBD and LBD, with other members of NRs. The DBD in NRs is characterized by two cysteine-rich zinc finger motifs, which are required for DNA binding and dimerization of NRs. The LBD contains sites for co-activator and co-repressor interactions, and mediates nuclear localization [31, 32]. In recent decades, ONRs have been actively characterized in their important regulatory roles involved in numerous key cellular processes and diseases, including cancer and also exploited as potential therapeutic targets for diseases mainly because of the presence of the “*druggable*” LBD [33, 34].

We have previously reviewed the emerging roles of ONRs in the development of prostate cancer. In particular, some ONR members (including RORγ, TR2, TR4, COUP-TFII, ERRα, DAX-1 and SHP) exhibit multiple cross-talks with AR signaling in both normal and malignant prostatic cells, indicating their intricate interplay in prostate cancer progression [35]. We also surveyed the expression profiles of the entire NR superfamily in 3D-cultured prostate cancer stem- or progenitor-like cells (PCSCs) and castration-relapse xenografts (VCaP-CRPC), and identified some ONRs (including RORβ, TLX, COUP-TFII, NURR1 and LRH-1) that show significant common up-regulation in 3D-cultured PCSC-enriched prostatospheroids and CRPC xenografts [24]. Over the years, a number of studies have gained significant advancement and understanding on the roles of ONRs (including RORγ [36], TR4 [37], TLX [38], ERRα [39, 40], SF-1 [41], LRH-1 [23], GCNF [42]) in CRPC progression. Their characterized roles in CRPC are summarized in Table 1.

**Table 1:** The expressions and characterized functions of orphan nuclear receptors in CRPC.

Gene names	Common names	Expression patterns in prostate cancer	Characterized functions and mechanisms in CRPC	Selective modulators	References
*NR1F3*	RORγ	Up-regulation in primary prostate cancer and further elevated in metastatic CRPC	Recruits nuclear receptor coactivators 1 and 3 to an AR-ROR response element (RORE) to stimulate *AR* gene transcription	SR2211, XY018, XY011	[36]
*NR2C2*	TR4	Higher expression in CRPC patients who failed to achieve PSA response than those who achieved PSA response	Not characterized	Retinol, retinoic acid	[37]
*NR2E1*	TLX	Up-regulation in metastatic hormone-refractory prostate cancer as compared to BPH and primary prostate cancer tissues	Represses *AR* transcription via recruitment of histone modifiers HDACs and LSD1	ccrp1,2 and 3, BMS453, All-trans retinal (ATRAL)	[38]
*NR3B1*	ERRα	1. Significant higher expression in prostatic tumors than that in benign foci and positively correlated with the Gleason score 2. Up-regulation in metastatic CRPC as compared to primary prostate cancer tissues	1. Forms a reciprocal loop with ERG to regulate the *TMPRSS2:ERG* fusion gene 2. Promotes intratumoral androgen synthesis via its direct transactivation of distinct set of steroidogenic enzyme genes	XCT790, Compound 29 (C29)	[39, 40]
*NR5A1*	SF-1	Significant higher expression in aggressive prostate cancer xenografts and cells.	Promotes intratumoral androgen synthesis via its direct transactivation of distinct set of steroidogenic enzyme genes	AC-45594, SID7969543, SID7970631	[41]
*NR5A2*	LRH-1	Up-regulation in hormone-naïve or neoadjuvant-treated prostate cancer than normal-adjacent tissues and further increased in CRPC tissues	Promotes intratumoral androgen synthesis via its direct transactivation of distinct steroidogenic enzyme genes	ML-180	[23]
*NR6A1*	GCNF	Up-regulation in hormone sensitive prostate cancer compared to normal prostate and further increased in metastatic lesions and CRPC	Not characterized	Unknown	[42]

## Orphan nuclear receptors-mediated intratumoral androgen biosynthesis in CRPC

Multiple nuclear transcription factors, including ONRs, are found to participate in the transcriptional regulation of steroidogenic enzyme genes expressed in endocrine tissues [43]. Recent studies from us and other group show that the ONRs can contribute to the castration-resistant growth of prostate cancer through their promotion of intratumoral androgen biosynthesis via front-door and/or backdoor pathways by their transcriptional regulation of multiple key steroidogenic enzyme genes [23, 40, 41].

Liver receptor homolog-1 (LRH-1, Ftz-F1, *NR5A2*), first cloned from a mouse liver cDNA library, belongs to the NR5A subfamily of NR superfamily. Although classified as an ONR, crystallographic studies show that some phospholipids can bind to LRH-1 and modulate its interplay with co-regulators and thus its transactivation [44]. LRH-1 is expressed at moderate to high levels in fetal and adult organs of endodermal origin (liver and intestines), steroidogenic organs (adrenal gland), gonads as well as adipose tissue, and exerts critical roles in the development and differentiation of endodermal organs and gonads, bile acid homeostasis, cholesterol metabolism, and reproduction [45]. LRH-1 has also been implicated in the tumorigenesis of multiple cancers, including breast cancer [46], pancreatic cancer [47], colon cancer [48], liver cancer [49], as well as ovarian epithelial and granulosa cell tumors [50]. Intriguingly, LRH-1 is identified as a critical regulator of steroidogenesis via it direct transcriptional regulation of multiple steroidogenic enzyme genes (e.g., *STAR, CYP11A1, HSD3B2, CYP17A1* and *CYP19A1*) in different steroidogenic organs (adrenal, testis and ovary) and non-steroidogenic tissues (adipose tissue) [45, 51, 52]. More importantly, LRH-1 can promote breast carcinogenesis by increasing the local estrogen production in adipose stroma surrounding breast carcinomas via its transactivation of the aromatase gene (*CYP19A1*) [53], suggesting indirectly that LRH-1 may play a positive role in the development of prostate cancer that is also influenced by the microenvironment containing sex steroids. Our recent study shows that LRH-1 displays an increased expression pattern in clinical CRPC tissues, CRPC xenograft models, and also abiraterone-treated CRPC tumors, and its overexpression can promote both in vitro androgen deprivation-resistant and in vivo castration-resistant growth capacities in AR-positive prostate cancer cells via its direct transactivation of multiple key steroidogenic enzyme genes (including *STAR, CYP11A1, HSD3B2, CYP17A1*) and enhanced intratumoral de novo production of androgens (T and DHT) in a CYP17A1-dependent manner. Notably, the resistance of prostate cancer cells to androgen-deprivation can be attenuated either by RNAi-mediated knockdown of LRH-1 expression, or by pharmacological suppression of LRH-1 activity with a LRH-1-specific inverse agonist ML-180 [23], suggesting that targeting LRH-1 could be a valuable therapeutic strategy approach for CRPC management.

Steroidogenic factor 1 (SF-1, AD4BP, *NR5A1*), another orphan member of NR5A subfamily, exhibits a high homology in structure with LRH-1; and functionally these two ONRs often bind to the same or highly similar response elements in their target genes [54]. As its name implies, SF-1 is a critical driving factor of steroidogenesis and functions of normal endocrine tissues, and acts as a key transcription factor to regulate the expression of genes responsible for cholesterol metabolism and conversion of steroid hormones [55, 56]. Previous studies reveal that SF-1 performs similar actions as LRH-1 in rat granulosa cell steroidogenesis [57], and its expression is associated with the aberrant cell growth in adrenocortical and ovarian cancers [58, 59]. Another study shows that SF-1 is essential for the FSH and cAMP signaling cascades to regulate aromatase gene (*CYP19A1*) and its interaction with β-catenin is responsible for estrogen production in ovarian granulosa cells [60]. More recently, Lewis et al. report that SF-1 can promote the aggressive growth of CRPC by stimulating steroid biosynthesis and cancer cell proliferation [41]. Their results show that SF-1 expression is absent in benign prostatic cells but present in aggressive prostate cancer cell lines. The presence of SF-1 affects progesterone production and induces the expression of certain steroidogenic enzyme genes, including *CYP17A1, HSD3B1, HSD17B3, and CYP19A1*. Moreover, SF-1 is sufficient and necessary to promote prostate cancer cell growth and proliferation and also mediate the growth of BCaPT10 prostate cell xenografts within a steroid-depleted environment [41]. Strikingly, the first synthetic SF-1 inverse agonist (AC-45594) is identified through Receptor Selection and Amplification Technology, and it is characterized to potently inhibit SF-1 activity and lead to the down-regulation of some key SF-1 target genes, including enzymes critical to steroid biosynthesis [61]. In the same year, through a rational high-throughput screening approach, two analogous isoquinolinones SID7969543 and SID7970631 are identified by another research group, as potent, selective and cell-penetrant inhibitors of SF-1 [62]. These studies implicate that targeting SF-1 may represent a potential therapeutic strategy for patients with CRPC.

Estrogen-related receptor α (ERRα, NR3B1, *ESRRA*) is an orphan member of the NR3B subfamily and is named due to its high homology with estrogen receptor α (ERα, *ESR1*). Studies in past decades indicate that ERRα, together with its coactivators PGC-1s (α or β), is the master transcriptional regulator in multiple bioenergetics pathways and mitochondrial functions, including glucose metabolism, mitochondrial oxidative metabolism and biogenesis [63]. Previously, we and others show that ERRα displays an up-regulation pattern in advanced prostate cancer, and is closely linked to its poor clinical and pathological outcomes [39, 64, 65]. Functional and mechanistic studies indicate that ERRα can promote the hypoxic growth adaptation of prostate cancer cells via its stabilization and augmentation of HIF-1 signaling [66], and also can form a reciprocal regulatory loop with an oncogenic transcription factor ERG on the regulation of *TMPRSS2:ERG* fusion gene and thus to promote the malignant growth of prostate cancer [39]. Interestingly, Seely et al. demonstrate that ERRα can promote the production of adrenal steroids by regulating *SULT2A1* that catalyzes the sulfonation of DHEA to more stable DHEA sulfate (DHEA-S) [67]. Moreover, ERRα is characterized to increase the local estrogen production by up-regulating aromatase (CYP19A1) expression in response to prostaglandin E2 in prostate stromal cells [68]. These findings suggest that ERRα can modulate the intracellular steroidogenic capacity, which prompts us to determine the functional roles of ERRα in intratumoral androgen biosynthesis in prostate cancer. As expected, our recent study reveal that ERRα, which exhibits an up-regulation expression pattern in metastatic CRPC tissues and also the castration-relapse VCaP-CRPC xenograft model, can function to promote the castration-resistant growth of prostate cancer by enhancing the intratumoral androgen biosynthesis and thus activation of AR signaling via a mechanism of direct transactivation of two key androgen biosynthesis enzyme genes *CYP11A1* and *AKR1C3* [40]. In addition to enhancing de novo androgen synthesis from cholesterol via its regulation of *CYP11A1*, ERRα can also increase intratumoral DHT biosynthesis via the secondary backdoor pathway (DHEA → → 5α-Adione → DHT) by directly targeting the *AKR1C3*. More importantly, inhibition of ERRα activity by its inverse agonist XCT790 or Compound 29 (C29), not only can attenuate the intratumoral androgen production and suppress AR signaling in prostate cancer cells, but also significantly prevent the castration-relapsed tumor growth accompanied with a decrease of intratumoral DHT production and reduced expression of AKR1C3, suggesting that targeting ERRα could be a potential ADT strategy for CRPC management [40].

**Fig. 2:**
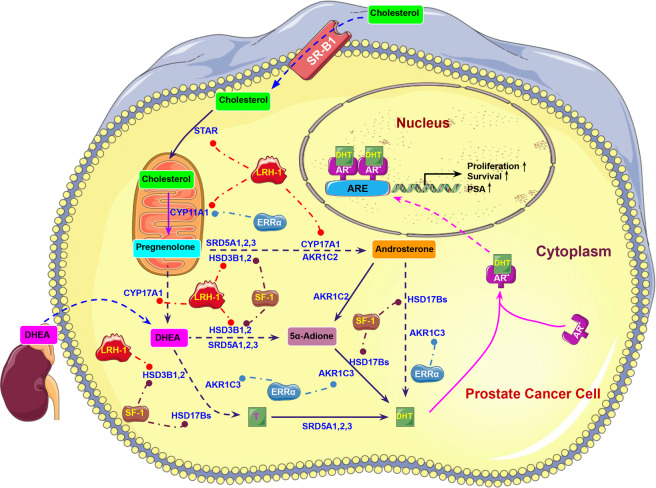
Schematic diagram illustrates the specific roles of ERRα, LRH-1, and SF-1 in the regulation of key enzymatic genes involved in intratumoral androgen biosynthesis in prostate cancer cells. Multiple key enzymatic genes involved in distinct pathways of androgen biosynthesis can be transcriptionally activated by ERRα, LRH-1, and SF-1; and their transactivations contribute to the increase of intratumoral androgen concentration in prostate cancer cells, resulting in activation of AR signaling and thus fueling the castration-resistant growth of prostate cancer cells.

Together, these findings represent a novel paradigm of regulation of intratumoral androgen biosynthesis in CRPC, in which prostate tumor cells may switch over to alternative pathways of androgen production and buffer themselves from the insufficiency of systemic androgens following ADT via different ONR-mediated transcriptional regulation of steroidogenic enzymatic machinery. It is worth noting that the three ONRs can target both common (e.g., *CYP17A1* by LRH-1 and SF-1, *CYP11A1* by LRH-1 and ERRα) and specific steroidogenic enzyme genes, and therefore be involved in distinct pathways of androgen biosynthesis in prostate cancer, in which ERRα and SF-1 participate in both front-door and backdoor pathways of DHT biosynthesis, whereas LRH-1 is responsible for the similar pathways except the secondary backdoor pathway. These results indicate that different ONRs may perform either differential or synergistic roles in the biosynthesis of DHT via their regulation of different or common steroidogenic enzyme genes involved in the intratumoral androgen biosynthesis in CRPC. Therefore, targeting the druggable ONRs could be a valuable therapeutic strategy approach for CRPC treatment. The working model of ERRα, LRH-1 and SF-1 in intratumoral androgen biosynthesis in prostate cancer cells is illustrated in Fig. 2.

In addition to the three reported ONRs with implicated functions in intratumoral androgen biosynthesis in prostate cancer, several other orphan members of NR superfamily have been characterized to be involved in steroidogenesis in certain non-prostatic organs or tissues, suggesting that these ONRs may also be the potential regulators in intraprostatic or intratumoral androgen biosynthesis in prostate cancer and yet to be further characterized [69]. COUP-TFII (*NR2F2)*, an ONR of the highly conserved NR2F subfamily, displays an increased expression pattern in advanced prostate cancer, and its expression is positively correlated with prostate tumor recurrence and progression [70–72]. One study performed in the mouse Leydig cells reveals that COUP-TFII can promote androgen production by directly transactivating the expression of *STAR* that is responsible for the cholesterol transport within the mitochondrial matrix, the first and the key step for steroidogenesis. In addition, COUP-TFII can cooperate with SF-1 on the activation of *STAR* gene promoter, due to the overlaps of binding elements between COUP-TFII and SF-1 [73]. However, some opposite observation was noted, in which the COUP-TF family members (COUP-TFI/*NR2F1* and COUP-TFII/*NR2F2*) are proposed as negative regulators of steroidogenesis in bovine glomerulosa cells [74]. Therefore, the regulatory roles of COUP-TFII in steroidogenesis may be likely tissue- or species-type dependent. Similarly, Nurr77 (*NR4A1*), an orphan member of the NR4A subfamily, which is highly expressed in advanced prostate cancer and characterized to promote prostate cancer invasion following ADT [75], can control the *STAR* transcription in mouse Leydig cells in either SF-1-dependent or -independent manner [76, 77]. Lastly, two atypical members of the NR superfamily without the classical zinc-finger containing DBD, DAX-1 (*NR0B1*) and SHP (*NR0B2*), also play regulatory roles in steroidogenesis, generally acting as the co-regulators of SF-1 or LRH-1. DAX-1 is frequently regarded as a negative regulator of steroidogenesis, where it can repress the LRH-1-mediated transcription of *HSD3B2* (3βHSD2) in ovarian granulosa cells [78], and also inhibit SF-1-mediated transcription of genes involved in androgen biosynthesis (*STAR, CYP11A1*, and *CYP17A1*) in adrenal cells [79, 80]. In regard to SHP, previous studies show that deficiency of SHP results in higher circulating T levels in SHP-knockout mice due to enhanced expression of steroidogenic genes *STAR* and *CYP11A1*, which is mediated by LRH-1 [81]; and besides, SHP can also repress the expression of *CYP17A1* by preventing the binding of LRH-1 to its promoter in liver cells [82].

## Concluding remarks and future perspectives

Intratumoural androgen biosynthesis represents a key mechanism driving the castration-resistant progression in advanced prostate cancer. Some key steroidogenic enzymes, which are upregulated in CRPC and hence supporting the relapse growth of CRPC, have become the emerging therapeutic targets for CRPC with a therapeutic purpose to reduce the intraprostatic androgen levels. Current treatment for metastatic and therapy-resistant CRPC with the CYP17A1 inhibitor, abiraterone acetate, is initially effective but still cannot stop the lethal progression of the disease. Thus, targeting the upstream factors involved in the regulation of steroidogenic enzymes and exploration of the mechanisms by which intratumoral androgen biosynthesis is initiated and maintained may represent a potential novel strategy for the management of CRPC. Over the years, studies on ONRs have gained sufficient insights to our current understanding on their roles in prostate cancer advancement, particularly CRPC. Given their important roles in the transcriptional regulation of steroidogenesis as well as CRPC, studies shown by us and others reveal that three distinct ONRs, including ERRα, LRH-1 and SF-1, can contribute to the CRPC progression by promoting the intratumoral androgen biosynthesis through their positive and differential transcriptional regulation of multiple critical steroidogenic enzyme genes involved in both the front-door and backdoor pathways of androgen biosynthesis. Although the endogenous ligands for ONRs have not yet been identified, more and more synthetic compounds working as agonists, inverse agonists or antagonists for ONRs have been developed recently (refer to Table 1). It is worthy of further studies to identify or develop novel ONRs modulators (natural or synthetic compounds) as the potential therapeutic agents for better management of CRPC.
